# Scrub Typhus Incidence Modeling with Meteorological Factors in South Korea

**DOI:** 10.3390/ijerph120707254

**Published:** 2015-06-29

**Authors:** Jaewon Kwak, Soojun Kim, Gilho Kim, Vijay P. Singh, Seungjin Hong, Hung Soo Kim

**Affiliations:** 1 Forecast and Control Division, Nakdong River Flood Control Office, Busan 604-851, Korea; E-Mail: firstsword@korea.kr; 2 Columbia Water Center, Columbia University, New York, NY 10027, USA; 3 Department of Hydro Science and Engineering, Korea Institute of Civil Engineering and Building Technology, Goyang-si, Gyeonggi-do 411-712, Korea; E-Mail: kgh0518@kict.re.kr; 4 Department of Biological & Agricultural Engineering and Zachry Dept. of Civil Engineering, Texas A & M University, TX 77843, USA; E-Mail: vsingh@tamu.edu; 5 Department of Civil Engineering, Inha University, Incheon 402-751, Korea; E-Mail: hongsst81@gmail.com (S.H.); sookim@inha.ac.kr (H.S.K.)

**Keywords:** scrub typhus, ANN, meteorological variables

## Abstract

Since its recurrence in 1986, scrub typhus has been occurring annually and it is considered as one of the most prevalent diseases in Korea. Scrub typhus is a 3rd grade nationally notifiable disease that has greatly increased in Korea since 2000. The objective of this study is to construct a disease incidence model for prediction and quantification of the incidences of scrub typhus. Using data from 2001 to 2010, the incidence Artificial Neural Network (ANN) model, which considers the time-lag between scrub typhus and minimum temperature, precipitation and average wind speed based on the Granger causality and spectral analysis, is constructed and tested for 2011 to 2012. Results show reliable simulation of scrub typhus incidences with selected predictors, and indicate that the seasonality in meteorological data should be considered.

## 1. Introduction

Scrub typhus is a rickettsial disease caused by intracellular bacteria *Orientia tsutsugamushi* and is transmitted by infection to humans through various species of infected Trombiculidae mites that feed on lymph and tissue fluid [1]. It is widely known as an endemic disease in Japan and is widely distributed within a 13 million km^2^ area of Southeast Asia and the Pacific Rim regions and approximately 1 billion persons are estimated at risk of the disease [2]. In South Korea (hereinafter referred to as Korea), it was first reported in 1951 and it reappeared in 1986 [3]. Since then scrub typhus incidences have been reported every year. Now it is considered as one of the most prevalent diseases in the southwestern provinces of Korea [4]. Generally, vector-borne diseases are transmitted by arthropods which can be greatly affected by climate [5]. Considering the results of Choi [6], who suggested that meteorological characteristics in Korea appear to have actually changed from 2000 onwards, it can therefore be inferred that scrub typhus is also affected by climate change.

Kalra and Rao [7] claimed that scrub typhus occurred in Kashmir, India, in a relatively temperate climate. There are many studies that have contributed to the knowledge on how scrub typhus is related to meteorological factors and is forecasted. The seasonal occurrence of scrub typhus varies according to climate in different countries [1], and the disease is found to occur more commonly during rainy season [8,9]. Kasuya [10] investigated the relationship between scrub typhus and meteorological factors using regression analysis. Kawamura *et al.* [11] analyzed the relationship between scrub typhus and climate type which reflects the behavior and population of Trombiculidae. Also, Zhang *et al*. [12] analyzed the relationship of scrub typhus and meteorological factors by regression analysis in Shijiazhuang City, China. The seasonality of scrub typhus suggests that meteorological variables might influence the spread of the disease [13].

Since climate change is now accepted as real, this change affects infectious disease pathogens and agents that influence the duration of infection, disease distribution [14,15] and spread to various vector and rodent-borne diseases [16,17,18,19,20]. Hence, recent studies have focused on determining the correlation between scrub typhus and meteorological factors and their influence on the disease [1,21,22,23,24,25,26,27]. Li *et al.* [28] found annual mean, maximum and minimum temperature and precipitation values to be correlated with scrub typhus, and Li *et al.* [13] showed that scrub typhus and monthly temperature, duration of sunshine, and rainfall were positively associated. Kim and Jang [25] also showed that temperature and humidity were closely correlated with scrub typhus in Korea. Kuo *et al.* [29] have reported a higher risk for scrub typhus infection in the endemic area with a higher normalized difference vegetation index (NDVI) [30] in Taiwan. Especially, Yang *et al.* [31] showed that the temperature with time-lag is important for the scrub typhus occurrence. However, very few studies have been completed on simulating or predicting the incidences with meteorological factors.

The objective of this study, therefore, is to investigate the incidence of scrub typhus and its correlation with meteorological factors and to construct a model, which employs Artificial Neural Network (hereinafter referred to as ANN), for incidences in Korea. By simulating the scrub typhus incidences in Korea, based on observed meteorological factors, the model can provide basic data of disease control for public health agencies. For this study, data on monthly scrub typhus occurrences and meteorological factors from 2001 to 2012 were collected. The constructed model was tested for 2011 and 2012 and the trend of incidences and seasonality were analyzed.

## 2. Scrub Typhus in Korea

### 2.1. Incidence Trend of Scrub Typhus

Since the reappearance of scrub typhus in 1986, cases of incidences have remarkably increased [32]. Especially, the occurrence of scrub typhus has significantly increased from 2000 onwards and it is continuously increasing. For example, 238 patients were reported in 1994, 4698 cases in 2004, and 10,365 cases in 2013, showing a 24% increase annually after 2000 onwards and it is now considered as one of the most prevalent diseases affecting humans in southwestern provinces of Korea [4]. Infection through *Leptotrombidium* (hereinafter referred to as *L. scutellare* and *L. pallidum*) is the reported cause of the scrub typhus in Korea, and *L. akamushi* hasn’t been reported yet [33], *L. scutellare* is the major cause of scrub typhus in Korea [34].

Scrub typhus is transmitted to humans through larvae bites of trombiculid mites and its habitat is located in low trees and bushes [35]. The mites that carry scrub typhus are affected by climatic conditions during the life-cycle [11]. Therefore, in Korea, an increase of scrub typhus infection is strongly related to the change in meteorological conditions [36,37] caused by climate change [38].

### 2.2. Data Collection

For this study, monthly data of the designated infectious diseases between 2001 and 2012, from the Center for Disease Control & Prevention [33], were utilized for obtaining data on the scrub typhus incidences of whole Korea. Data from 2001 to 2010 were used for calibration and data from 2011 to 2012 were used for validation. Also, meteorological data were obtained from the Korea Meteorological Administration (KMA). But the geographical distribution of Trombiculidae, which is mainly responsible for scrub typhus, is concentrated in the southwestern province of Korea [39].

**Figure 1:**
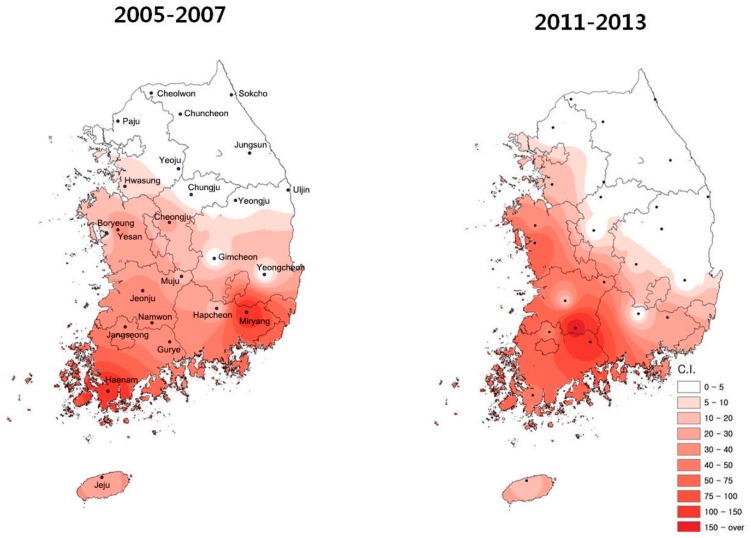
Geographical distribution of Orientia tsutsugamushi vector, *L. scutellare* density in Korea [39]; C. I. (confidence interval) color and number indicated the collected cases of chiggers that has scrub typhus (*L. scutellare*).

**Figure 2:**
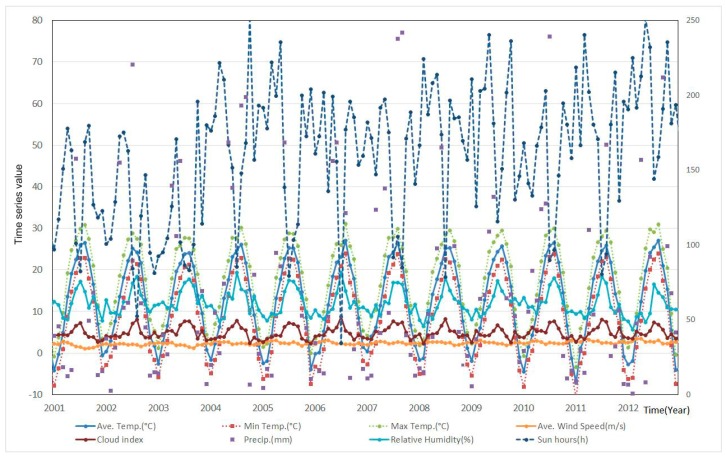
Weighted average meteorological data from Korea [44].

**Figure 3:**
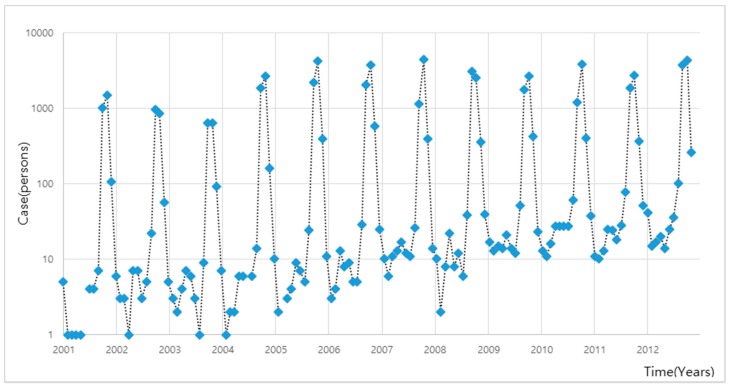
Scrub typhus incidence cases in Korea [33].

We collected 24 meteorological observatory (2001 to 2007) and 25 meteorological observatory (2008 to 2013) meteorological data, and these data were weighted averaged into one, which considered the spatial distributions (C.I. number of *L. scutellare* in Figure 1) of *L. scutellare* as in study by [39]. Meteorological data included in the analysis were monthly average, maximum and minimum air temperatures (°C), precipitation (mm), relative humidity (%), wind speed (m/s), duration of sunshine (hours), and cloud amount, which are known as responsible factors for scrub typhus [31,40,41,42]. Also, some studies have indicated that land use affects scrub typhus [29], but Jin *et al.* [43] showed the land use changes did not affect Scrub typhus. Hence, it was not taken into account in this study. The collected meteorological data are shown in Figure 2 and scrub typhus in Figure 3.

## 3. Methodology

Many studies consider meteorological factors, which are related to epidemic diseases, as a statistical variable, not a time series, but Yang *et al.* [31] suggested scrub typhus displayed a time-lag effect, and Kim and Jang [25] showed that scrub typhus had a correlation with meteorological factors in the summer season in Korea. Therefore, this study considered meteorological and scrub typhus data as time series. When doing so, we employed the Granger causality and cross-spectral analysis to determine meteorological factors that have correlation with scrub typhus and time-lag, and then constructed a scrub typhus incidence model with an artificial neural network.

### 3.1. Granger Causality

Granger [45] suggested the idea of causality as a tool to determine that variable (or time series) whereby X causes variable Y if knowing X helps predict the future of Y. This is referred to as the Granger causality whereby X can be identified as the cause of Y when the inclusion of past observations of X reduces the prediction error of Y in a linear regression model, as compared to a model which includes only previous values of Y. Therefore, the Granger causality has been widely employed in many fields that need to determine the cause one among the set of unclear variables [46]. The Granger causality can be described by a bivariate autoregressive model as:

$$\text{X}\left(\text{t}\right)=\;\overset{k}{\underset{i=1}{\sum }}A_{1}X\left(t-i\right)+\;\overset{k}{\underset{i=1}{\sum }}A_{2}Y\left(t-i\right)+\;\;\epsilon_{1}\left(t\right)$$

(1) $$\text{Y}\left(\text{t}\right)=\;\overset{k}{\underset{i=1}{\sum }}B_{1}Y\left(t-i\right)+\;\overset{k}{\underset{i=1}{\sum }}B_{2}X\left(t-i\right)+\;\;\epsilon_{2}\left(t\right)$$

where X and Y are each time series, t denotes the index of time step in the time series, k is the maximum number of lag in each time series, A and B are the model parameters of each time series, and ϵ_1_ and ϵ_2_ are the error measurements of model. If the variance of ϵ_1_ or ϵ_2_ is reduced by the inclusion of Y or X, it is evident that X is the cause of Y or Y is the cause of X. In this case, A or B is significantly different from zero and it can be estimated by the logarithm of the corresponding F-statistic [47].

### 3.2. Cross Spectrum and Wavelet Spectrum

Autospectrum or cross spectrum is a method to explain the distribution of correlation (or cross-correlation) or variance of frequency drawn from a single or multiple data [48]. It is useful to investigate the changing frequency of meteorological data [49]. Autocorrelation function can be obtained by dividing the autocovariance by σ^2^, the variance of X(t). Autospectral function ${\text{X}}_{\text{X}}\;\left(\text{n}\right)$ can be obtained by applying the Fourier transform to the autocorrelation function (n is the frequency of data), so autospectral analysis gives periodic information of each time series. Like the autospectrum, cross spectral analysis can also be applied to cross-covariance of two time series X(t), Y(t) and can be also used to differentiate the relevant covariance of two time series data set to have lag k. Cross spectral function ${\text{X}}_{\text{xy}}(n$) of two times series can be determined using Equations (2) and (3):

(2) $${\text{VAR}}_{\text{X}}\left(\text{k}\right)=\;\frac{1}{\text{N}-\text{k}-1}\;\overset{\text{N}-\text{k}}{\underset{\text{t}=1}{\sum }}\left({\text{X}}_{\text{t}}-\overset{\text{X}}{¯}\right)\left({\text{X}}_{\text{t}+\text{k}}-\overset{\text{X}}{¯}\right)$$

(3) $${\text{X}}_{\text{XY}}\left(n\right)=\;\overset{\text{N}}{\underset{\text{k}=0}{\sum }}\left[\frac{{\text{COV}}_{\text{XY}}\left(\text{k}\right)}{{\text{σ}}_{\text{X}}{\text{σ}}_{\text{Y}}}\right]{\text{e}}^{\frac{\text{i }2\text{π f k}}{n}}$$

where N is the number of data and t and ∆t are the time step and time interval in the each time series, and k denotes the time lag. So, cross-spectrum gives correlation as a frequency function and it can be used to determine the phase difference between two time series. Also, using the cross- spectrum on two time series, coherency can be calculated and information about the frequency between the two (see Equation (4)) can be obtained. Coherency between two time series is defined as:

(4) $${\text{C}}_{\text{xy}}\left(\text{n}\right)=\;\frac{{\left|{\text{X}}_{\text{xy}}\left(n\right)\right|}^{2}}{{\text{X}}_{\text{x}}\left(n\right){\text{ X}}_{\text{y}}\left(n\right)}$$

where ${\text{C}}_{\text{xy}}$ is the magnitude-squared coherence, and ${\text{X}}_{\text{x}}{\text{ and X}}_{\text{y}}$ are the autospectral densities of the two time series. The coherence value will always satisfy $0<C_{\text{xy}}<1$ and it can be used to estimate the causality between the input and output when the data are ergodic and the system function is linear [50].

Also, wavelet spectrum (transformation) is the method to expand time series into time frequency space and it can be used to find localized intermittent periodicities [51]. Especially, continuous wavelet transform (CWT) is desirable to examine two time series that may be expected to be linked in some way [52]. The CWT of the time series is defined as:

(5) $$W_{X}\left(\text{s},\text{t}\right)=\text{ X}\left(\text{t}\right)\;\times \;\varphi_{s}\left(\text{t}\right)$$

(6) $$\varphi_{s}\left(\text{t}\right)=\;\pi^{-1/4}\;\times \;e^{i\omega t}\times \;e^{\frac{t^{2}}{2}}$$

where $\varphi_{s}\left(\text{t}\right)$ is the Morlet wavelet [53] with scale s and the power of wavelet can be defined as ${\left|{W_{n}}^{X}\left(\text{s}\right)\right|}^{2}$. Cross wavelet transform with two time series X(t), Y(t) can be defined as $W_{XY}\left(s,t\right)=\;W_{X}\left(\text{s},\text{t}\right)\;{W_{Y}}^{*}\left(s,t\right)$ and asterisk denotes complex conjugation, so we further define the cross wavelet power as $\left|W_{XY}\right|$. This complex argument arg ($W_{XY}$) can be interpreted as the local relative phase between two time series X(t), Y(t) in the time frequency space [52].

### 3.3. Artificial Neural Network

Kihoro *et al.* [54] have shown that ANNs are better than Autoregressive Integrated Moving Average (ARIMA) models in their forecasting ability for seasonal time series, so we employed an ANN for scrub typhus forecasting. The ANN [55] mimics the structure and functions of a biological neural system in which neurons are connected through nodes [56]. Since the perceptron was proposed to categorize information patterns [57], ANNs have been widely used to recognize nonlinear relationships between different variables. The ANN is composed of three layers: the input layer represents observed meteorological data, the output layer produces simulated incidence cases as a result of network and the hidden layer is constituted of a network of neurons (non-linear functions) that are trained to recognize patterns of observations.

**Figure 4:**
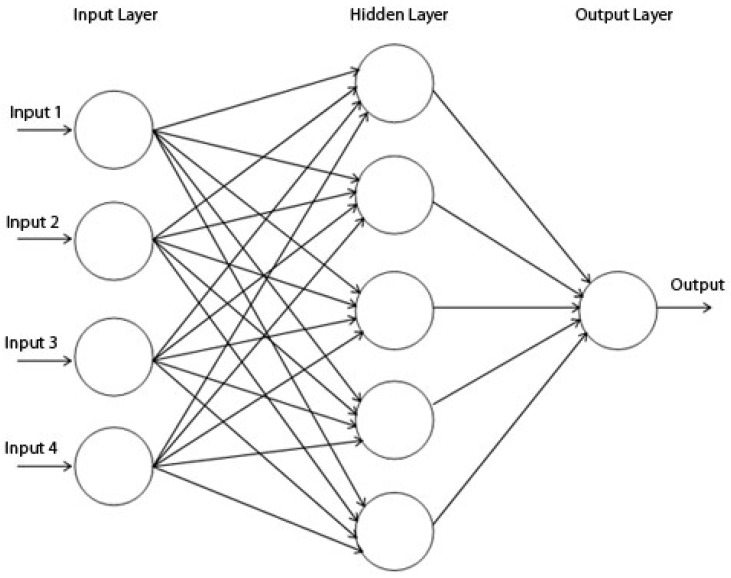
ANN schematization [58]. *Input i* is the input set, *output* is the result of network delay and each circle represents neural network.

The back-propagation algorithm has been used to train the network [59]. The Levenberg-Marquardt-QNBP algorithm has been used to optimize the parameters of the network and it is known to produce good results for non-linear problems, such as related meteorological data [59,60,61].

## 4. Scrub Typhus Modeling with Meteorological Factors

### 4.1. Predictor Selection and Construction of Incidence Model

**Figure 5:**
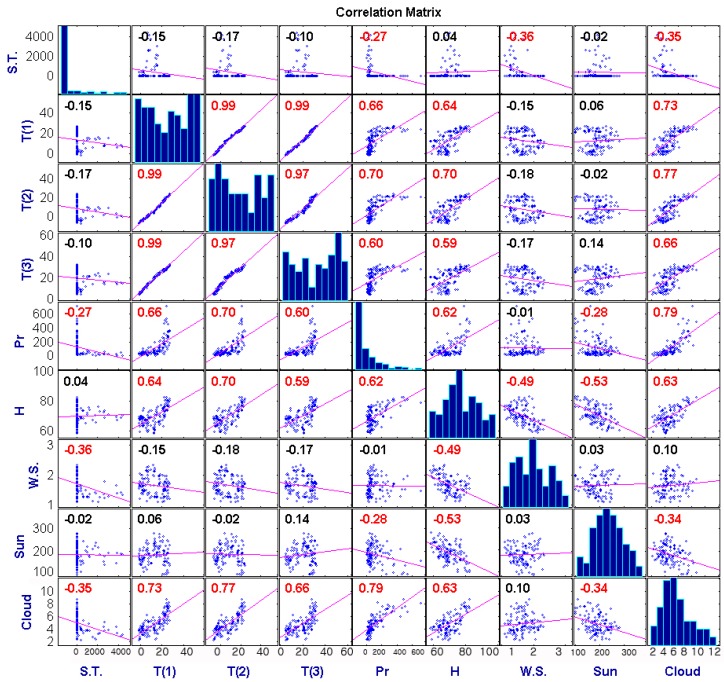
Scatter plot of scrub typhus and each variable, the number indicates Pearson’s correlation coefficient **:** S.T; Scrub typhus, T(1); mean temperature (°C), T(2); minimum temperature (°C), T(3); maximum temperature (°C), Pr.; precipitation (mm), H; relative humidity (%), W.S.; mean wind speed (m/s), Sun; duration of sunshine (hours), Cloud; cloud amount, and red marks indicate that the correlation coefficient has a statistically significant meaning, and the histogram is a non-dimensional plot.

Many previous studies [25,62,63] show that scrub typhus and meteorological data in Korea have seasonality. Strictly, it may be due to error in analysis [64], as Briët *et al.* [65] showed that in most cases strong correlation between malaria and precipitation that is considered most highly correlated with many epidemic diseases in Sri Lanka was spurious. Therefore, monthly mean value of 20 years was subtracted from the values of each meteorological factor used in this study. This method is widely used to remove seasonality [66,67]. Figure 5 shows the correlation matrix between scrub typhus and each meteorological data set which was detrended.

As a result of Figure 5, it seems that it is hard to find suitable meteorological factors to construct a model to estimate scrub typhus incidences. Kim and Jang [25] reported that scrub typhus is highly correlated with meteorological conditions in the summer season. Therefore, we employed the Granger causality and cross-spectral analysis to determine what the proper predictor and time-lag (*k*) between scrub typhus and meteorological factors are. As Song *et al.* [68] and Kim and Jang [25] show that scrub typhus in Korea mainly spawns in the summer season, the Granger causality was calculated for one to six month time lags and detrended meteorological factors, and results are shown in Table 1.

**Table 1:** Granger causality result with scrub typhus and detrended meteorological factors.

Meteorological Factor	Number of Lag (*k*)					
	1	2	3	4	5	6
Mean Temp. (°C)	61.74 **	3.60	1.62	3.87	6.02 *	6.06 *
Min. Temp. (°C)	4.01	0.96	1.96	3.55	7.02 *	7.60 *
Max. Temp. (°C)	89.86 **	14.77 **	12.14 *	5.85	5.18	5.32
Precipitation (mm)	3.67	4.00	4.61	4.19	4.03	6.14 *
Relative Humidity (%)	1.37	3.73	5.38	7.81 *	9.35 *	8.26 *
Wind Speed (m/s)	1.68	1.97	2.11	29.11 **	40.10 **	43.06 **
Duration of sunshine (h)	3.61	3.23	5.43	3.90	3.10	5.10
Cloud amount	4.37	4.68	5.53	4.44	3.90	4.15

* and ** indicate statistically significance level at p=0.1 and p=0.05.

**Figure 6:**
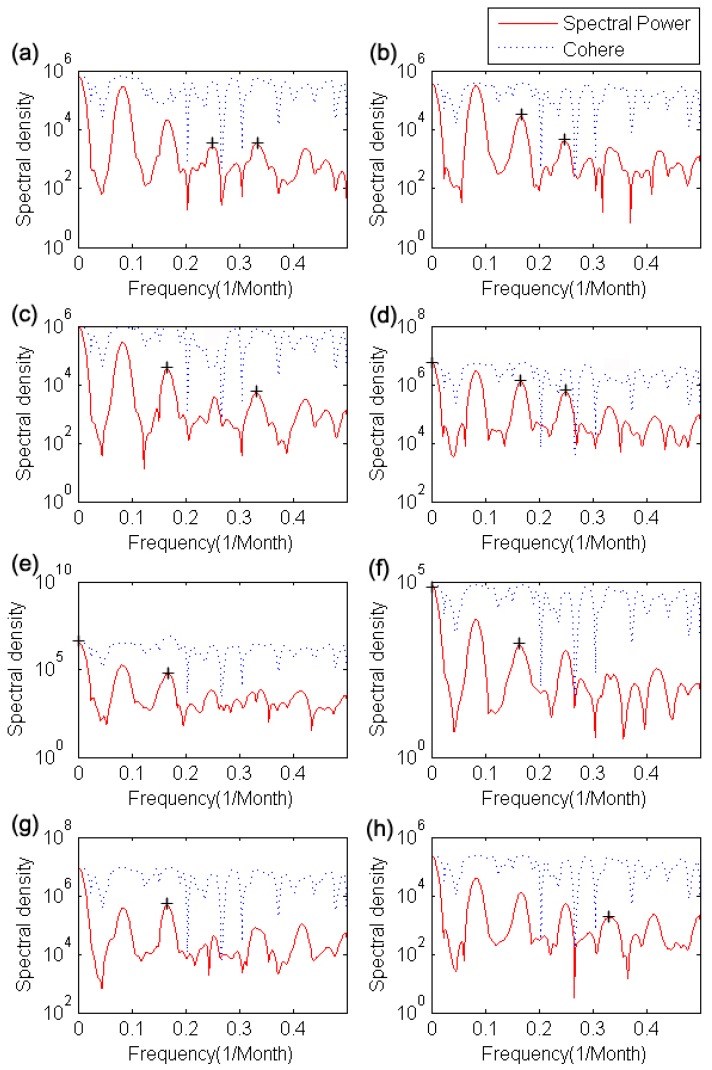
Cross spectrum and coherence of scrub typhus and each meteorological factor: scrub typhus with (**a**) mean temperature; (**b**) minimum temperature; (**c**) maximum temperature; (**d**) precipitation; (**e**) relative humidity; (**f**) wind speed; (**g**) duration of sunshine; and (**h**) cloud cover, all coherence results are up-scaled for visibility, and each cross spectrum (red line) and coherence (blue dot line) indicate that the correlation power and its explanation power, and + mark indicate that the frequency (1/f) has correlation power between scrub typhus and each meteorological factor.

As shown in Table 1, scrub typhus is strongly correlated with mean temperature (1 month lag), maximum temperature (1 and 2 month lags), and average wind speed (5 and 6 month lags), and moderately correlated with mean temperature, minimum temperature, precipitation and relative humidity, but the duration of sunshine and cloud cover are not correlated. This result is similar to that of previous studies in Korea [25,40,69,70]. We also conducted cross-spectral analysis for scrub typhus and meteorological factors to determine a reasonable time-lag for input variables, as shown in Figure 6.

Also, cross-spectral and coherence analysis indicate that scrub typhus is correlated with several meteorological factors which have time-lag (k). But the spectral density at ${\text{X}}_{\text{XY}}\left(0.083\right)$ shows that scrub typhus has its own seasonal frequency, so we neglected ${\text{X}}_{\text{XY}}$ and ${\text{C}}_{\text{xy}}$ at the 12 month lag. Finally, we selected several meteorological factors (mean and maximum temperatures with  k=1, 3, 4 and k=1, 3, 6; minimum temperature with k=3, 6 precipitation with k=1, 4, 6; and relative humidity and wind speed with k=1, 6), based on Granger’s causality and cross spectrum and coherence value, as input predictors that take account of the results of Table 1 and Figure 6.

**Figure 7:**
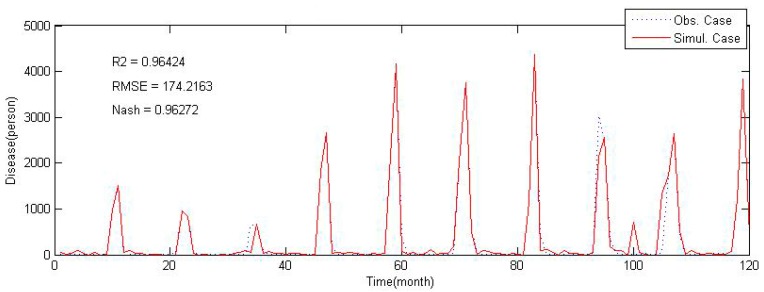
Calibration results of scrub typhus incidence model.

We constructed a scrub typhus incidence model based on ANN with selected predictors. The ANN of this study has 5 hidden layers which were optimized by trial and error [71]. The calibration results of ANN are shown in Figure 7. To compare model results with observed values, R^2^ [72], Root Mean Squared Error (RMSE) [73], and Nash-Sutcliffe efficiency coefficient [74], which are widely used to evaluate models, were employed. Calibration results of the constructed scrub typhus incidence model showed R^2^ of 0.96, RMSE of 174, and the Nash coefficient of 0.96. These evaluation measures are certainly greater than those in other studies that even showed some negative fitted values [75] or R^2^ of 0.689 [10].

### 4.2. Validation of the Incidence Model

**Figure 8:**
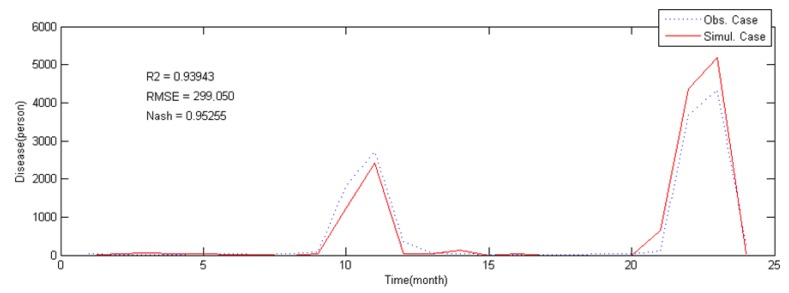
Validation result of scrub typhus incident model.

The scrub typhus incidence mode based on ANN, was evaluated using the incidence data from 2011 to 2012, as shown in Figure 8.

Results of evaluation showed R^2^ of 0.94, RMSE of 299, and the Nash-Sutcliffe coefficient of 0.95. Both the calibration and the validation results indicate that selected predictors are mainly responsible in the scrub typhus modeling. The evaluation results of the whole period are shown in Table 2.

**Table 2:** Evaluation function value of each phase.

E. Func. Period	R^2^	Nash	RMSE (Person)
Calibration (2001–2010)	0.946	0.963	174
Validation (2011–2012)	0.939	0.953	299

## 5. Results and Discussion

This study was conducted for constructing a scrub typhus incidence model with the use of ANN. For this, we selected proper predictors with time-lag (*k*) from among meteorological factors based on the Granger causality and cross spectral analysis. As shown in Table 1 and Figure 6, scrub typhus is mainly correlated with meteorological factors with 1 month and 4 to 6 month time lags. For example, the mean temperature is correlated when k=1, 3, 4 months, minimum temperature is when k=3, 6, maximum temperature is when k=1, 3, 6, precipitation is when k=1, 4, 6, and relative humidity and wind speed are when k=1, 6. Generally, the occurrence of scrub typhus depends on temperature, because its vectors, Trombiculidae mites, are ectothermic insects. Therefore, time lag (k=1) correlation between scrub typhus and each temperature are not surprising, because Trombiculidae mite activity depends on temperature. But there are other times lags (k=3, 4 and 6) for temperature, precipitation, relative humidity and wind speed that seem to be related with the spawning condition. In Korea, Trombiculidae are known to mainly spawn in summer and stop spawning during fall and winter seasons [76] and Trombiculidae spawning rates are increasing with temperature [77]. In particular, the adult Trombiculidae stop their spawning when humidity is low [76]. Therefore, a large time-lag (k=3, 4 and 6) indicates spawning in summer season (k=3, 4 and 6 correspond to May, July and August). The wind speed also shows some correlation with scrub typhus, but wind speed was not considered in the previous studies on epidemiology and ecology. Hence, there are no clues as to why or how it has correlation with the scrub typhus occurrence, and further studies are needed in the perspective of ecology of the Trombiculidae life cycle or infection mechanism with wind speed. With this and validation results (Figure 7 and Figure 8) in mind, the proposed incidence model can be used for short-term forecasting of scrub typhus incidences in Korea.

The second thing is the seasonality of meteorological data. The seasonality in the meteorological factors was removed before using them as predictors, otherwise it can cause error in the incidence model predictions. However, there is uncertainty that seasonality will really cause error in the constructed model. Because Granger’s causality or 1 dimensional spectral analysis cannot detect seasonality and correlation, we performed cross-wavelet analysis to visualize these characteristics in time-domain spaces. Following Torrence and Compo [78] and Grinsted *et al.* [52], we used wavelet transform and selected the frequency scale factor.

**Figure 9:**
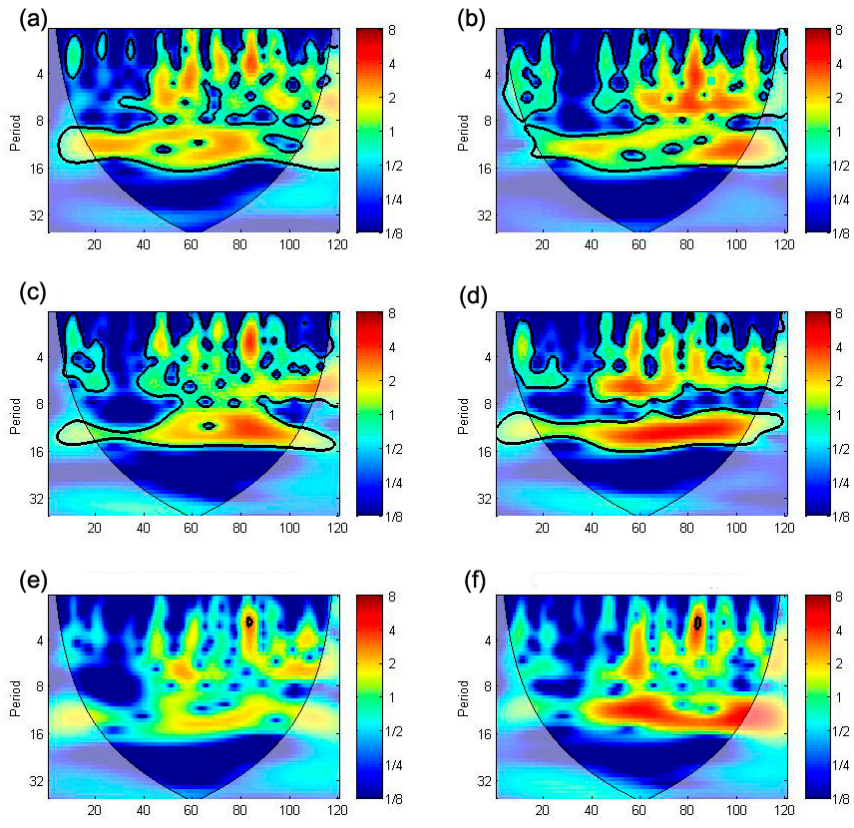
Cross wavelet analysis results between scrub typhus and processed meteorological predictors (removed seasonality): scrub typhus with (**a**) mean temperature; (**b**) precipitation; (**c**) relative humidity; (**d**) wind speed; (**e**) duration of sunshine; and (**f**) cloud amount; bold line indicates 95% confidence inbound which is statistically significant and white transparency area indicates un-confidence area, and blue to red colors indicate the temporal correlation scale with each color-map index.

**Figure 10:**
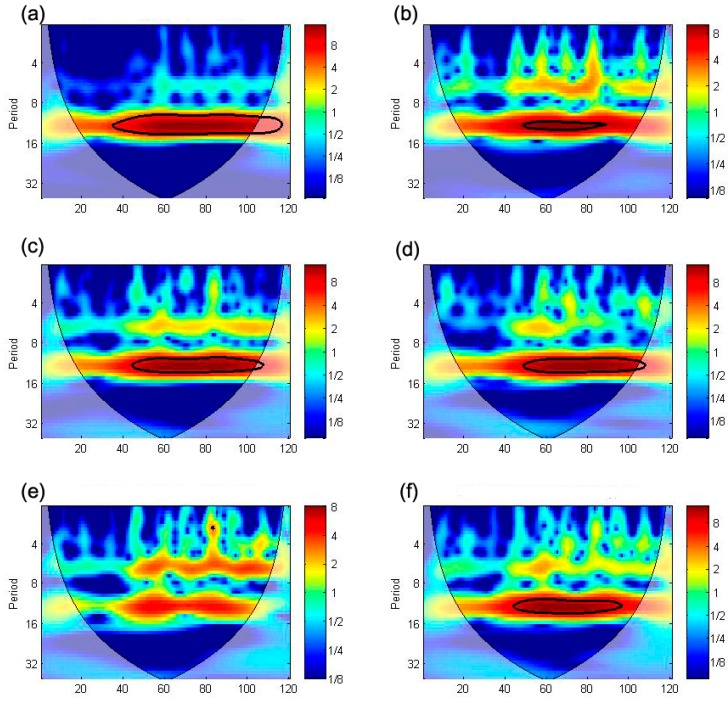
Cross wavelet analysis between scrub typhus and raw meteorological predictors (non-removed seasonality): scrub typhus with (**a**) mean temperature; (**b**) precipitation; (**c**) relative humidity; (**d**) wind speed; (**e**) duration of sunshine, and (**f**) cloud cover; bold line indicates 95% confidence inbound which is statistically significant and white transparency area indicates un-confidence area, and blue to red colors indicate the temporal correlation scale with each color-map index.

Meteorological predictors that have seasonality show 11 to 13 month (approximatively 12 month) cycle correlation for the whole period with 95% confidence inbound, as the bold lined area in Figure 10, but they do not have significant correlation with scrub typhus outbreak events. This means that scrub typhus has its own occurrence cycle and it is weakly correlated with meteorological predictors, which also have their own seasonality (Figure 10), so meteorological data cannot be used as predictors. However, several studies show that meteorological factors are most responsible for scrub typhus in Korea. On the other hand, Figure 9 shows correlation for each year outbreak time with mean temperature, precipitation, relative humidity, and wind speed with 1 to 7 months lag. This strongly suggests that 11 to 13 month seasonality in raw meteorological predictors (Figure 10) can cause spurious correlation, which is mainly affected by the seasonality of meteorological data [79], which are used in the correlation analysis or regression, or even in the network models. Therefore, the study on Scrub typhus modeling, relating to meteorological factors, in Korea should better consider the seasonality of meteorological data.

A limitation of this study is the lack of long time incidence data. Scrub typhus incidence data from 2001 to 2012 years were collected for this study. These 12 years data may suffice to construct the incidence model, but it is difficult to prove if the data are really enough. The incidence of scrub typhus in Korea has significantly increased from average 250 cases per year to average 1,300 cases per year in 1997 to 1998 year and has stabilized at average 3500 cases per year near 2001 to 2005 for [33]. Also, meteorological conditions have actually changed from 2000 onwards [6]. So, the use of data before the 2001 year may cause error in the study. Accordingly we obtained incidence data for 2001 to 2012. Also, the method suggested in this study is clearly a way to construct a prediction model for epidemic diseases which are correlated with meteorological factors. Especially, recent studies suggested that the future meteorological data can be acquired through the climate model with 3 month lead time [80], with maximum 18 month lead time for ENSO [81]. Therefore, the suggested method and model can provide reliable data, which are outbreak time and approximate case number based on meteorological data for the decision maker or agencies of public health.

## 6. Conclusions

The study clarifies the correlation between monthly meteorological data and scrub typhus incidence and establishes a reliable incidence ANN model. It also shows that the seasonality of meteorological factors affects model prediction in Korea. From the results of this study the following conclusions may be drawn:

(1) A scrub typhus incidence model with an ANN model is constructed. Based on the correlation between scrub typhus cases and monthly meteorological data, the mean, maximum and minimum temperatures, precipitation, relative humidity, wind speed data were selected as predictors. Also, appropriate time-lags were selected using Granger’s causality and cross spectrum and coherence. The constructed model is validated from 2011 to 2012 and R^2^ is 0.94, RMSE is 299 and Nash efficiency is 0.95, which clearly account for scrub typhus incidence. So, the method and the incidence model suggested in this study can provide reliable data for the decision makers or agencies of public health.
(2) To visualize the seasonality effect in the predictors, cross-wavelet analysis is conducted. Meteorological predictors without seasonality eliminated show a strong 11 to 13 month correlation cycle during the whole period. But the results for predictors with seasonality removed show 1 to 7 month correlation for each year outbreak time with mean temperature, precipitation, relative humidity and wind speed. This suggests that seasonality can affect correlation. This means that any scrub typhus prediction model in Korea should consider seasonality.
